# GLP-1 Receptor Agonists in Mood Disorders: A Psychiatric Perspective

**DOI:** 10.3390/life15091422

**Published:** 2025-09-10

**Authors:** Pietro Carmellini, Alessandro Cuomo, Maria Beatrice Rescalli, Andrea Fagiolini

**Affiliations:** Department of Molecular and Developmental Medicine, Division of Psychiatry, School of Medicine, University of Siena, 53100 Siena, Italy

**Keywords:** GLP-1 receptor agonists, mood disorders, insulin resistance, personalized psychiatry

## Abstract

Mood disorders, including major depressive disorder (MDD) and bipolar disorder (BD), are among the leading causes of disability worldwide and are frequently associated with treatment resistance, functional impairment, and high comorbidity with metabolic dysfunction. Increasing evidence implicates insulin resistance (IR) as a key pathophysiological factor linking metabolic and psychiatric illness. IR is associated with chronic low-grade inflammation, hypothalamic–pituitary–adrenal (HPA) axis dysregulation, impaired neuroplasticity, mitochondrial dysfunction, and altered reward processing mechanisms that may contribute to core depressive features such as anhedonia, cognitive slowing, and emotional dysregulation. These processes are further exacerbated by the metabolic side effects of many psychotropic medications, creating a self-perpetuating cycle that worsens both psychiatric and physical health outcomes. Glucagon-like peptide-1 receptor agonists (GLP-1 RAs), initially developed for type 2 diabetes and obesity, have emerged as promising candidates to address this metabolic–psychiatric interface. Beyond improving glycemic control and promoting weight loss, GLP-1 RAs exert central actions relevant to mood disorders, including modulation of dopaminergic reward pathways, enhancement of hippocampal neurogenesis, attenuation of neuroinflammation, and regulation of appetite and energy balance. Preclinical studies demonstrate that GLP-1 RAs reduce microglial activation, promote hippocampal neurogenesis, and normalize stress-induced behavioral changes. Early clinical trials in patients with metabolic disorders suggest improvements in depressive symptoms, quality of life, and cognitive function, with some effects independent of weight loss or glycemic outcomes. Observational evidence also indicates reduced antidepressant use and psychological distress in diabetic and obese populations receiving GLP-1 RAs. While these findings are promising, large randomized controlled trials in primary psychiatric populations are lacking. Key challenges include clarifying dose–response relationships, disentangling central from peripheral effects, and addressing safety and adherence concerns in individuals with comorbid psychiatric conditions. Future research should focus on biomarker-informed stratification, comparative trials with standard treatments, and integration of GLP-1 RAs into multimodal care frameworks. Overall, GLP-1 RAs represent a biologically plausible and clinically relevant approach to bridging metabolic and psychiatric care, with the potential to improve outcomes in patients with mood disorders who carry a high metabolic burden.

## 1. Introduction

Mood disorders, including major depressive disorder (MDD) and bipolar disorder (BD), remain a major cause of disability worldwide, with lifetime prevalence exceeding 15% for depression and up to 3% for bipolar disorder, with more than 400 million people affected by depression [1]. These conditions are often persistent, associated with reduced functional capacity, and respond only partially to available treatments. Even with recent advances in pharmacotherapy, many individuals continue to experience symptoms, particularly anhedonia, cognitive slowing or fatigue, and emotional instability [2], and up to one-third of patients develop treatment resistance [3]. These limitations highlight the urgent need for innovative approaches that integrate metabolic and psychiatric dimensions to improve outcomes and quality of life.

Increasing evidence implicates insulin resistance (IR) as an important factor in the pathophysiology of mood disorders [2,4,5]. IR is frequently accompanied by chronic, low-grade systemic inflammation, driven by elevated concentrations of pro-inflammatory cytokines such as TNF-α, IL-6, and IL-1β, produced by adipose tissue and immune cells in the context of metabolic dysfunction [6,7]. This inflammatory state not only disrupts metabolic regulation but also impacts brain function. Circulating cytokines can cross the blood–brain barrier and activate microglia, which subsequently release additional inflammatory mediators, including IL-1, IL-6, and TNF-α. Neuroinflammation in turn, interferes with neuroplasticity by reducing neurogenesis, altering synaptic architecture, and dysregulating neurotransmitter systems [7,8], changes that are closely linked to mood disturbance, cognitive impairment, and affective lability in individuals with IR [4,6,9].

Alterations of the hypothalamic–pituitary–adrenal (HPA) axis, a hallmark of stress-related psychiatric disorders, are also more pronounced in patients with insulin resistance [2,9]. Hyperglycemia and pro-inflammatory cytokines can exacerbate this dysregulation, contributing to heightened stress reactivity and mood instability [10]. Several studies describe a bidirectional association between IR, HPA axis dysfunction, and depression, particularly among patients with type 2 diabetes [5,9]. Hyperinsulinemia, the defining metabolic feature of IR, may further drive both metabolic and affective disturbances. Notably, comorbid mood disorders appear more prevalent and severe in people with IR, with a particularly strong link observed in obese adolescents with neuroendocrine abnormalities [11,12].

In addition to inflammatory and neuroendocrine mechanisms, IR has been associated with mitochondrial dysfunction and impaired brain energy metabolism, particularly in regions central to mood regulation, such as the prefrontal cortex and limbic system [2,4,6]. Adding to these vulnerabilities; many psychotropic drugs have adverse effects on metabolic health; increasing the risk of obesity; type 2 diabetes; and non-alcoholic fatty liver disease [13,14,15]. This underscores the complex, bidirectional relationship between psychiatric and metabolic disorders.

It is important to acknowledge, however, that the relationship between insulin resistance and mood disorders is likely bidirectional. Depression itself can contribute to metabolic dysregulation through mechanisms such as physical inactivity, altered dietary patterns, sleep disruption, and chronic stress physiology, all of which can promote insulin resistance. Lifestyle factors, therefore, act both as confounders and as mediators, complicating the directionality of causality. This emphasizes the need for longitudinal and mechanistic studies that resolve the cause and consequence of the relationship between IR and depression.

In this context, glucagon-like peptide-1 receptor agonists (GLP-1 RAs) have emerged as potential therapeutic agents. Initially developed for type 2 diabetes and obesity, these drugs also exert central effects relevant to mood regulation, including modulation of reward circuits, appetite control, and attenuation of neuroinflammation [8,16,17]. Early studies suggest that GLP-1 RAs may reduce depressive symptoms, either via direct neurobiological effects or indirectly through improvements in metabolic health and body weight [18].

This narrative review examines the potential application of GLP-1 RAs in mood disorders, with a particular focus on their dual effects on insulin resistance and mood-related symptoms. We outline the biological mechanisms linking IR and mood dysregulation, discuss the metabolic consequences of psychotropic medications, and summarize the current evidence for GLP-1 RAs in psychiatric populations. Finally, we consider the clinical implications of integrating metabolic-targeted strategies into psychiatric care, advocating for a more individualized, metabolically informed approach.

## 2. Materials and Methods

The purpose of this narrative review is to synthesize the current theoretical and empirical evidence on the role of glucagon-like peptide-1 receptor agonists in mood disorders, with a particular focus on their impact on insulin resistance and affective symptoms. We aimed to integrate findings from multiple domains, including psychiatry, endocrinology, and neurobiology, to provide a comprehensive overview of potential translational applications.

A literature search was conducted in the electronic databases PubMed and Scopus up to July 2025. The search strategy included combinations of MeSH terms and free-text terms such as “GLP-1 receptor agonists,” “insulin resistance,” “major depressive disorder,” “bipolar disorder,” “mood disorders,” “neuroinflammation,” and “reward system.” Articles published in English were considered, and we included clinical trials, observational studies, systematic and narrative reviews, meta-analyses, preclinical animal studies, and cellular models relevant to GLP-1 RAs in the context of psychiatric and metabolic disorders. Case reports, editorials, and non-peer-reviewed material were excluded.

Reference lists of eligible publications were also screened to identify additional studies. Given the narrative design, no formal systematic assessment of study quality was undertaken; instead, we prioritized peer-reviewed research that directly addressed psychiatric or mechanistic outcomes of GLP-1 receptor agonists.

## 3. Psychiatric Relevance of GLP-1 Signaling

Glucagon-like peptide-1 receptor agonists have shown effects that extend beyond glucose regulation, impacting several neurobiological systems involved in mood disorders [15,19]. At the peripheral level, they support glycemic control and insulin sensitivity by promoting glucose-dependent insulin release from pancreatic β-cells, inhibiting excess glucagon secretion, and slowing gastric emptying [20,21]. These combined actions lead to improved postprandial glucose control, reduce appetite, and lead to weight loss, outcomes that directly oppose insulin resistance, a condition frequently seen in individuals with mood disorders and known to worsen psychiatric prognosis [2,22].

In the brain, GLP-1 receptors are present in regions critical for mood and motivation, including the hypothalamus, prefrontal cortex, ventral tegmental area (VTA), and nucleus accumbens [23]. Among these, the arcuate and paraventricular nuclei of the hypothalamus, as well as the VTA and nucleus accumbens, exhibit particularly high receptor density [24]. Activation of hypothalamic GLP-1 receptors enhances satiety signaling by influencing neurons that express pro-opiomelanocortin (POMC) and neuropeptide Y (NPY), mechanisms that reduce excessive food intake and the metabolic load tied to obesity [17,24]. In addition, GLP-1 RAs appear to modulate dopamine pathways within mesolimbic circuit networks that play a key role in processing reward and motivation. This effect may be particularly relevant in individuals with depression marked by anhedonia or poor treatment response [25].

Importantly, GLP-1 RAs also possess anti-inflammatory and neuroprotective properties, which are increasingly seen as relevant in the context of mood disorders [15,26]. Several preclinical studies have shown that these compounds reduce central neuroinflammation by lowering levels of pro-inflammatory cytokines (e.g., IL-1β, IL-6, TNF-α) and by limiting oxidative stress [27,28]. Since these processes are linked to impaired neurogenesis and synaptic plasticity, their modulation may support neuronal health and resilience, particularly in stress-related mood symptoms. While anti-inflammatory and neuroprotective effects of GLP-1 receptor agonists have been described, it remains unclear to what extent these benefits are attributable to peripheral metabolic improvements (e.g., weight loss, improved insulin sensitivity) versus direct central actions on brain circuits. The relative contribution of these mechanisms likely varies across patients, depending on their metabolic status. The ability of GLP-1 RAs to penetrate the blood–brain barrier and mediate central effects (e.g., appetite suppression) can differ based on the molecular structure of the agonist and individual patient characteristics such as blood–brain barrier permeability, obesity, and metabolic health. Additionally, the magnitude of peripheral metabolic effects, like insulin secretion, glucagon suppression, and modulation of gut hormones, can also be influenced by a patient’s existing metabolic status and degree of insulin resistance [29,30].

Overall, the combined metabolic, neurochemical, and immunomodulatory effects of GLP-1 RAs suggest they could offer therapeutic benefits in mood disorders, especially for patients with coexisting metabolic dysfunction or resistance to standard treatments [15,19,20]. Their ability to address both physiological and psychiatric dimensions supports a more integrated view of mood disorders as systemic conditions, rather than disorders confined to brain circuits alone [31,32]

## 4. Mood Disorders and Metabolic Comorbidity: The Bidirectional Interface

Insulin resistance, long considered a defining feature of metabolic syndrome and a risk factor for cardiovascular disease, is increasingly recognized as a key contributor to the pathophysiology of mood disorders [21,33,34]. Far from being a consequence of poor lifestyle or medication side effects alone, IR has been detected in young, drug-naïve, and non-obese psychiatric patients, suggesting it may represent an intrinsic biological vulnerability in affective illness [12,35].

Numerous studies have documented a high prevalence of IR in individuals with major depressive disorder and bipolar disorder, even in the absence of obesity or overt metabolic disease [36,37]. A large meta-analysis of over 240,000 participants found elevated insulin levels and HOMA-IR indices in those with acute depression, independent of antidepressant use and body weight [22]. Similarly, cross-sectional studies in drug-naïve BD patients have shown a significantly higher risk of IR compared to healthy controls, with no correlation to BMI or comorbidities [12,38]. These findings challenge the view of IR as merely a metabolic byproduct and instead position it as a potential core component of mood pathology [34].

This has led to the identification of a “metabolic subtype” of depression, marked by IR and clinical features such as anhedonia, low energy, cognitive slowing, and poor response to first-line antidepressant treatments [25,33]. These symptoms persist even after controlling for physical activity and BMI, pointing to an independent role for metabolic dysfunction in shaping affective symptomatology. IR has also been identified as a predictor of non-response to selective serotonin and noradrenaline reuptake inhibitors (SNRIs), further reinforcing its association with treatment-resistant depression [35].

Neurobiological studies support this link, showing that IR is associated with structural and functional brain changes, including reduced volumes in regions involved in mood regulation and cognition. These alterations may underlie the clinical features of cognitive slowing and diminished reward sensitivity observed in patients with IR [39,40]. Additionally, higher levels of IR predict slower improvement in symptoms such as anhedonia during antidepressant treatment and are associated with worse longitudinal outcomes, including increased relapse risk and hospitalization [35,41,42]. For example, Watson et al. found that even mild increases in IR over a nine-year period were associated with an 89% greater risk of developing MDD [5].

Prospective epidemiological studies reinforce the relationship between metabolic dysfunction and mood disorders [21,43]. A large Dutch cohort study showed that moderate elevations in IR markers, such as increased waist circumference, fasting glucose, and elevated triglyceride-to-HDL ratios, were linked to a significantly higher risk of developing MDD over time [3]. Every 5 cm increase in waist circumference, for instance, was associated with an 11% greater risk of incident depression, and higher fasting glucose conferred a 37% increased risk. In that study, 14% of participants without prior depression developed MDD over nine years, with those exhibiting higher baseline IR measures at significantly higher risk [5].

IR appears to be particularly relevant in bipolar disorder, where its prevalence exceeds 50% in some samples [37,44,45]. Patients with BD and comorbid IR or type 2 diabetes are more likely to experience rapid cycling, a chronic course of illness, and poor response to lithium or other mood stabilizers [34,46]. IR in BD has been associated with neuroprogression, cognitive decline, and treatment resistance, independent of age, BMI, or exposure to antipsychotics [42,44].

Mechanistically, IR represents a critical biological interface between peripheral metabolic dysfunction and central nervous system changes. It contributes to low-grade inflammation, mitochondrial dysfunction, altered energy metabolism, impaired neuroplasticity, and dysregulation of the HPA axis [47,48]. Peripheral IR may also exacerbate central insulin resistance, disrupting dopaminergic signaling and further impairing brain function [49].

Compounding the issue, many commonly prescribed psychotropic medications, including second-generation antipsychotics, mood stabilizers, and certain antidepressants, are known to worsen metabolic parameters [50,51,52,53]. This creates a vicious cycle in which psychiatric symptoms and metabolic dysfunction reinforce one another, further complicating treatment outcomes [42].

Taken together, the evidence positions insulin resistance not only as a metabolic concern but also as a central player in the onset, severity, and treatment resistance of mood disorders. Understanding IR as a shared pathophysiological mechanism opens the door to more targeted, integrated approaches to treatment, particularly in patients with atypical features, poor therapeutic response, or evidence of metabolic dysregulation [54].

## 5. Psychotropic Medications and Metabolic Dysregulation

Psychotropic medications, including antipsychotics, mood stabilizers, and certain classes of antidepressants, are major contributors to metabolic dysfunction in individuals with mood disorders [55,56]. Among these, second-generation antipsychotics (SGAs) such as olanzapine, clozapine, and risperidone exhibit the highest metabolic liability [57,58,59]. These agents are known to induce weight gain, dyslipidemia, and IR through mechanisms that involve the antagonism of histaminergic (H1), serotonergic (5-HT2C), and muscarinic (M3) receptors [52]. This receptor profile interferes with hypothalamic appetite regulation, reduces energy expenditure, and impairs peripheral insulin signaling. (Table 1).

Mood stabilizers like lithium and valproate, although less potent in this regard, are also associated with increased weight and impaired glucose metabolism, especially with long-term use [50,53]. Certain antidepressants, particularly tricyclic antidepressants and some SSRIs (e.g., paroxetine), have been linked to modest increases in adiposity and reduced insulin sensitivity [51,60]. Crucially, these pharmacologically induced changes compound an underlying vulnerability: individuals with psychiatric disorders show a higher baseline prevalence of insulin resistance, metabolic syndrome, and type 2 diabetes, even in young, non-obese populations [45,57]. This predisposition is likely multifactorial, involving shared genetic, inflammatory, and neuroendocrine mechanisms [61].

Beyond physical health, IR appears to exert a detrimental effect on psychiatric outcomes [62]. Several studies have linked insulin resistance to residual depressive symptoms, particularly anhedonia, fatigue, and cognitive slowing, which are often resistant to standard antidepressant treatments [19,63,64]. Functional neuroimaging suggests that IR affects reward-related brain regions such as the ventral striatum, potentially mediating these symptoms [40,65,66]. This bidirectional relationship between psychotropic medications and metabolic dysfunction creates a self-reinforcing pathogenic cycle: psychotropic medications worsen insulin resistance, which contributes to reduced treatment efficacy, often leading to dose escalation or polypharmacy. This, in turn, intensifies the metabolic burden, further undermining both psychiatric and somatic health [55,67,68].

Disrupting this cycle is a clinical priority. Current guidelines recommend regular metabolic monitoring in patients on psychotropics, along with early implementation of lifestyle interventions [51,57]. Moreover, pharmacologic strategies such as metformin or GLP-1 receptor agonists are gaining interest not only for their metabolic benefits but also for their potential to improve mood and cognitive outcomes in patients with insulin resistance and treatment-resistant depression [62,69].

## 6. Clinical Studies in Psychiatric Populations

A growing body of clinical research suggests that GLP-1 receptor agonists may offer antidepressant benefits, particularly in individuals with comorbid metabolic disturbances [13,58]. Initial evidence has come from studies in patients with type 2 diabetes, where agents like liraglutide and semaglutide have not only improved glycemic control but also been associated with reductions in depressive symptoms [63,64,69]. In some trials, improvements in mood appeared independently of changes in HbA1c, pointing to potential neuropsychiatric effects that go beyond glycemic normalization [8,65,66].

This therapeutic effect has also been explored in patients with obesity and major depressive disorder, a population often affected by overlapping pathophysiological processes such as insulin resistance, inflammation, and altered reward system function [33,42,70]. Preliminary findings from both randomized controlled trials and observational studies suggest that treatment with GLP-1 RAs leads to significant weight loss and metabolic improvements, alongside decreases in depressive symptoms and enhancements in health-related quality of life [19,62]. Although sample sizes remain small, the consistency of results across studies underscores the potential of integrated metabolic-psychiatric approaches [41].

Weight reduction and improved insulin sensitivity may indirectly alleviate depressive symptoms by reducing systemic inflammation and restoring metabolic balance [21,43]. Modulation of leptin and adiponectin signaling, as well as a reduction in HPA axis overactivation, may further contribute to mood stabilization [47,48].

In addition, early preclinical and clinical studies have demonstrated direct central nervous system effects of GLP-1 Ras [71,72]. These actions have been associated with improvements in mood and cognition [39]. When used alongside standard antidepressant or antipsychotic treatments, GLP-1 RAs may offer additional benefit, particularly for patients with mood disorders and coexisting metabolic issues [34,42]. Psychotropic medications, especially second-generation antipsychotics and some mood stabilizers, are well known for their metabolic side effects, including weight gain, insulin resistance, and dyslipidemia. In this setting, GLP-1 RAs present a unique therapeutic option by targeting both residual mood symptoms and medication-induced metabolic burden [51,62,69].

By improving insulin sensitivity, promoting weight loss, and restoring glucose metabolism, GLP-1 RAs engage biological pathways relevant to both mood dysregulation and metabolic syndrome [8,63,72]. Their inclusion in psychiatric treatment plans could address an often-overlooked clinical need: managing both psychiatric symptoms and their metabolic comorbidities, which significantly affect long-term prognosis and quality of life. Table 2 summarizes characteristics and main findings of studies of GLP-1 Ras in the psychiatric population.

Although data in psychiatric populations are still limited, safety and tolerability findings are encouraging [15,36,54]. Reported side effects are largely gastrointestinal, such as nausea, vomiting, and appetite suppression, and tend to be dose-dependent and transient. However, long-term adherence remains a significant challenge. High dropout rates in obesity and diabetes trials highlight tolerability issues, which may be further compounded in psychiatric populations where comorbid anxiety, cognitive impairment, or poor illness insight can interfere with consistent medication use. Practical barriers such as injectable administration, cost, and access may also limit feasibility. These factors underline the need for careful patient selection, structured behavioral support, and real-world studies to assess persistence and tolerability in psychiatric contexts. Importantly, there is no evidence of increased risk for suicidality, psychosis, or affective destabilization, key concerns in mood disorders [73]. Cognitive impairment and sedation have also not been reported, supporting their suitability as adjunctive agents [39]. That said, careful monitoring remains essential, especially in patients with complex medical conditions or polypharmacy [37,44].

By complementing monoaminergic mechanisms with modulation of reward-related pathways and inflammatory tone, GLP-1 RAs may work synergistically with conventional antidepressants, particularly in subtypes characterized by anhedonia or treatment resistance [63,66]. Overall, integrating GLP-1 RAs into the management of mood disorders represents a biologically sound and clinically promising strategy. These agents have the potential to improve treatment outcomes, lower cardiometabolic risk, and enhance functional recovery by addressing both the metabolic and psychiatric components of illness [21,42,62]. Larger, long-term randomized controlled trials are still needed to define optimal patient profiles, dosing strategies, and long-term safety in psychiatric care.

**Table 2:** Characteristics and main findings of studies of GLP-1 RAs in the psychiatric population.

Study (Author/Year and Ref)	Study Design and Population	Intervention	Key Outcomes on Mood and Cognition	Notes
Mansur et al., 2017 [74]	A 4-week, open-label pilot; n = 19 adults with MDD or BD and executive dysfunction	Liraglutide 1.8 mg/day adjunctive	Improved executive function (Trail Making Test-B; composite cognition z-score). No significant change in mood scales	First proof-of-concept in a mood-disorder cohort; cognitive benefits independent of glycemic changes; small sample
Bezin et al., 2025 [73]	Nationwide case-time-control (France); n = 1102 suicide/attempt cases, with psychiatric subgroups	GLP-1 RAs (various)	No increased risk of suicide/attempt (OR 0.62); consistent across psychiatric-history subgroups	Strong real-world evidence of psychiatric safety, including high-risk populations
Ueda et al., 2024 [75]	Active-comparator cohort (Sweden and Denmark); n = 298,553 T2D patients	GLP-1 RAs vs. SGLT2i	No excess risk of suicide, self-harm, incident depression or anxiety	Large-scale pharmacoepidemiology confirming reassuring safety
Farr et al., 2016 [76]	Randomized, placebo-controlled crossover; n = 18 T2D	Liraglutide up to 1.8 mg/day	Reduced brain activation to palatable food cues (parietal cortex, insula, putamen)	Demonstrates central GLP-1R activity; relevant to reward and anhedonia pathways
van Bloemendaal et al., 2014 [77]	Randomized crossover with GLP-1R blockade; n = 48 obese ± T2D	Exenatide ± GLP-1R antagonist	Decreased food-cue responses (insula, amygdala, OFC); reduced caloric intake	Confirms central GLP-1R mediation of reward-circuit responses
Coveleskie et al., 2017 [78]	Double-blind crossover fMRI; n = 19 women (obese vs. lean)	Exenatide 5 μg SC	In obese women: increased connectivity (NTS–thalamus/hypothalamus); reduced hunger ratings	Suggests obesity moderates central GLP-1 signaling; implications for satiety/reward
Fanelli et al., 2025 [9]	UK Biobank; n = 30,919 with MDD	Insulin resistance (HOMA-IR, proxies)	IR+ patients had greater antidepressant resistance, longer treatment duration, and more severe profiles	Supports targeting metabolic dysfunction in depression
Li et al., 2024 [12]	Cross-sectional; n = 125 drug-naïve BD vs. 85 controls	Assessment of insulin resistance	BD patients had 2–3× higher prevalence of IR; IR linked to hypersomnia	Confirms IR as a core metabolic abnormality in BD
Watson et al., 2021 [5]	NESDA longitudinal cohort; n = 601, 9-year follow-up	Baseline IR markers (TG/HDL, glucose, waist)	IR predicted incident MDD (HR 1.3–1.9); prediabetes doubled risk	Strong prospective evidence linking IR and depression onset
Shomaker et al., 2010 [11]	Cross-sectional; n = 136 adolescents	Psychological symptoms + insulin sensitivity	Depressive symptoms were inversely correlated with insulin sensitivity	Provides early-life evidence of mood–metabolic coupling
Weiss et al., 2024 [38]	Retrospective chart review; elderly MDD/BD (n≈100)	Naturalistic follow-up with/without T2D	T2D associated with greater manic morbidity in BD and cognitive decline	Highlights adverse psychiatric impact of metabolic comorbidity

## 7. Mechanistic Hypotheses for Psychotropic Action

GLP-1 receptor agonists have demonstrated a range of central nervous system (CNS) effects that align with key neurobiological pathways implicated in mood regulation, motivation, stress responsiveness, and cognitive function [74,76,79]. Several interrelated mechanisms have been proposed to explain their potential psychotropic effects [80,81].

A leading hypothesis centers on the modulation of neuroinflammation. Chronic low-grade inflammation, characterized by elevated levels of cytokines such as TNF-α and IL-6, is frequently observed in patients with mood disorders and metabolic dysregulation [26,82,83]. Both preclinical and clinical studies suggest that GLP-1 RAs reduce systemic and central inflammation, potentially restoring immune homeostasis in brain regions critical for emotional regulation and reward processing, such as the prefrontal cortex and limbic structures [26,82,84]. Beyond a general reduction in inflammatory cytokines, GLP-1 RAs appear to exert targeted effects on key inflammatory pathways. Experimental models show that GLP-1 receptor activation inhibits NF-κB signaling, thereby reducing transcription of pro-inflammatory mediators including IL-1β, IL-6, and TNF-α. GLP-1 RAs also attenuate activation of the NLRP3 inflammasome, a central driver of neuroinflammation and microglial reactivity in depression [85]. In parallel, modulation of the JAK/STAT pathway has been observed, further dampening cytokine signaling and promoting an anti-inflammatory ambient [86].

Another key pathway involves neurotrophic signaling. GLP-1 RAs have been shown to promote the expression of brain-derived neurotrophic factor (BDNF), enhancing synaptic plasticity and neurogenesis, particularly in the hippocampus and prefrontal cortex [84]. These regions are consistently implicated in the pathophysiology of depression, and such neurotrophic effects may underlie symptom improvements in domains like anhedonia, fatigue, and cognitive slowing [74,84]. Furthermore, GLP-1 RAs exhibit neuroprotective properties observed in models of neurodegenerative diseases, including Alzheimer’s and Parkinson’s, which may extend to the treatment of psychiatric conditions [26,79].

Preclinical studies in rodent models further support these mechanisms. Chronic administration of exendin-4 or liraglutide has been shown to enhance hippocampal neurogenesis, reduce anxiety and depression-like behaviors, and attenuate neuroinflammatory cascades through downregulation of IL-6 and TNF-α [63]. At the cellular level, GLP-1 receptor activation promotes neuroprotective signaling via cAMP-CREB-BDNF pathways, reduces oxidative stress, and prevents apoptosis in neuronal cultures exposed to hyperglycemic or inflammatory conditions [84,87]. Additional rodent studies have confirmed that GLP-1 RAs reduce depressive- and anxiety-like behaviors via modulation of hippocampal BDNF and suppression of neuroinflammation [88,89,90]. Systematic reviews and meta-analyses further support improvements in mood outcomes, underscoring the translational potential of these agents even if the heterogeneity of the studies included was high [15,91].

Importantly, GLP-1 RAs influence central reward circuitry. They enhance dopaminergic tone in the mesolimbic pathway, particularly the ventral tegmental area and nucleus accumbens, thereby improving motivational salience and hedonic responsiveness [80,81,92]. This may help alleviate anhedonia, a core symptom of depression [83]. While GLP-1 RAs reduce food intake by dampening hyperactive reward responses to palatable cues, they appear to preserve general reward sensitivity, differentiating them from medications that blunt dopaminergic tone nonspecifically [81,92]. Enhancing central insulin signaling, especially in individuals with insulin resistance, may further normalize dopaminergic function and contribute to the reversal of anhedonic phenotypes [40,41].

GLP-1 RAs have also been shown to alter brain functional connectivity [78]. Neuroimaging studies report modulation of networks such as the dorsal default mode network (DMN), salience network, and visuospatial attention circuits [76,78]. Specific compounds (e.g., exenatide, liraglutide) differentially affect connectivity in regions like the hypothalamus, thalamus, nucleus tractus solitarius, and hippocampus [78,93]. These effects may contribute to observed improvements in mood and cognitive domains [74].

A final and increasingly relevant mechanism involves the gut–brain axis [94,95]. GLP-1 RAs modulate gut microbiota composition, promote vagal afferent signaling, and influence gut-derived neuropeptides such as ghrelin and peptide YY (PYY), all of which can impact CNS function [96,97,98]. For example, liraglutide has been shown to activate vagal pathways and reduce postprandial triglyceride levels via a gut–brain–liver reflex [96]. Additionally, chronic treatment with GLP-1 RAs increases the abundance of anti-inflammatory microbial taxa (e.g., Bacteroidetes), which has been associated with improvements in both mood and glycemic control [94,95,99]. Preclinical studies confirm that long-term GLP-1 RA administration enhances hippocampal neurogenesis and reduces anxiety- and depression-like behaviors in rodent models [84].

In addition, GLP-1 RAs play a central role in the regulation of appetite and feeding behavior, which may indirectly influence mood [76,77]. Central GLP-1R activation in the amygdala also suppresses food intake, indicating a direct CNS mechanism linked to mood regulation [100]. Moreover, GLP-1 receptors are expressed in the brainstem and hypothalamus, key regions involved in energy homeostasis [76,77]. Activation of these receptors, either endogenously via oral or intraduodenal nutrient stimuli or through pharmacological administration, activates vagal afferents, a neural relay known to suppress appetite and improve glucose metabolism. Experimental knockdown of GLP-1Rs in the vagal pathway increases food intake and reduces insulin secretion in animal models, emphasizing the functional importance of this circuit [97]. Additionally, GLP-1 RAs modulate gut peptides such as ghrelin and can enhance PYY secretion, reinforcing satiety signaling [98]. These effects on appetite regulation may also contribute to improvements in self-perception, energy levels, and overall psychological well-being, especially in individuals with comorbid obesity or binge-related behaviors [74,77].

Taken together, these pleiotropic effects, ranging from anti-inflammatory, neurotrophic actions to dopaminergic and gut-mediated modulation and appetite-regulating effects, support a multimodal model of GLP-1 RA activity relevant to psychiatric disorders [26,83]. Their pleiotropic actions may be particularly beneficial in mood disorder patients with metabolic dysregulation, treatment resistance, or reward-processing deficits [74,83].

**Figure 1:**
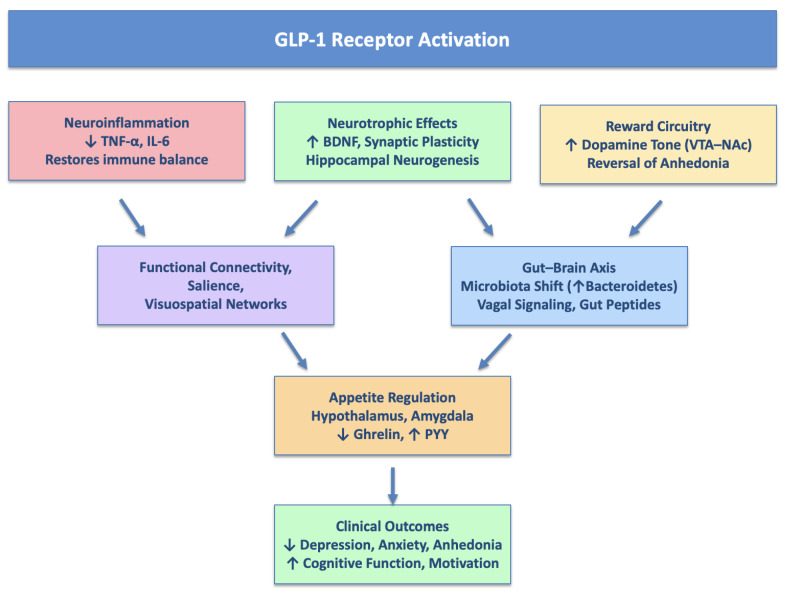
Main hypotheses for psychotropic action of GLP-1 Ras.

Figure 1 summarizes the main hypotheses for the psychotropic action of GLP-1 RAs.

## 8. Clinical Challenges and Research Priorities

Despite encouraging mechanistic and preliminary clinical findings, the integration of GLP-1 RAs into psychiatric treatment remains in an early and exploratory stage [18]. To date, most studies investigating their psychotropic potential have been conducted in populations with type 2 diabetes or obesity, where mood improvements may partially reflect metabolic normalization [101]. Consequently, the generalizability of these results to individuals with primary mood disorders, particularly those without overt metabolic disease, remains uncertain [18]. Large-scale, placebo-controlled randomized clinical trials specifically designed for psychiatric populations are critically needed to determine the efficacy, optimal dosing, duration, and long-term safety of GLP-1 RAs in treating mood symptoms [101]. Future trials should stratify patients by metabolic phenotype, incorporate mechanistic biomarkers, and include long-term follow-up to evaluate both psychiatric and metabolic endpoints. Comparative effectiveness studies with established antidepressants and mood stabilizers will also be essential. The integration of GLP-1 RAs into multimodal frameworks, including psychotherapy and lifestyle interventions, may represent a more effective precision psychiatry approach.

An additional challenge lies in the considerable metabolic heterogeneity observed among patients with mood disorders [102]. Individuals with MDD or bipolar disorder (BD) may vary widely in terms of insulin sensitivity, inflammatory status, and body composition, even in the absence of clinical metabolic syndrome [102,103]. This heterogeneity highlights the need for metabolic phenotyping in psychiatric research. Identifying biologically distinct subgroups, such as patients with central adiposity, insulin resistance, or elevated inflammatory markers, may enable the development of stratified treatment approaches, improving the likelihood of clinical benefit from GLP-1 RAs and similar agents [102].

Emerging data also point to a broader neuropsychiatric profile of GLP-1 RAs, with preliminary evidence suggesting benefits across multiple domains, including cognitive function, suicidality, anxiety, substance use, and binge-related eating behaviors. Some GLP-1 analogues may influence glutamatergic and GABAergic pathways, potentially contributing to therapeutic effects in conditions such as epilepsy, autism spectrum disorders, and addiction, although further validation is required. These findings underscore the potential for GLP-1 RAs to act on shared neurobiological substrates across psychiatric and neurological disorders [104,105].

Nevertheless, important safety considerations remain. Post-marketing surveillance and pharmacovigilance data have reported psychiatric adverse effects, including anxiety, mood lability, and suicidal ideation, in some cases, although these findings are agent-specific and not consistently observed in controlled studies [75,106,107]. Individualized risk–benefit assessment and clinical monitoring are warranted, particularly in vulnerable populations or those with complex psychiatric comorbidities. Although GLP-1 RAs are considered safe as monotherapy, their glucose-dependent mechanism of insulin secretion means that hypoglycemia is rare unless combined with insulin or sulfonylureas. Psychiatric patients with comorbid diabetes on such therapies require close monitoring. Currently, no specific clinical guidelines exist regarding the psychiatric use of GLP-1 RAs, highlighting the need for expert consensus and real-world implementation frameworks [18,103]. Current clinical studies have used standard antidiabetic or anti-obesity doses (e.g., liraglutide 1.8 mg/day; semaglutide 1.0–2.4 mg/week). Determining optimal doses for psychiatric indications, balancing efficacy against tolerability and metabolic safety, remains a key priority for future trials [15].

A promising avenue for improving precision in treatment selection involves the use of biomarker-informed strategies. Markers such as fasting insulin, HOMA-IR, leptin/adiponectin ratio, and high-sensitivity C-reactive protein (hs-CRP) may help identify patients with metabolic-inflammation-driven subtypes of depression or bipolar disorder [102,105]. Incorporating such markers into trial design and clinical workflows could enhance predictive accuracy and treatment responsiveness, though standardization and validation in prospective cohorts are needed.

Finally, translational studies aimed at disentangling the relative contributions of peripheral metabolic improvements versus central neuromodulatory effects (e.g., anti-inflammatory action, synaptic plasticity, dopamine regulation) are essential. These investigations will help clarify the mechanistic basis of GLP-1 RA efficacy in psychiatry and guide the development of next-generation incretin-based compounds with enhanced CNS specificity [102,103,105]. In parallel, future research should also address the integration of GLP-1 RAs into multimodal treatment frameworks, combining pharmacological, psychotherapeutic, and lifestyle-based interventions. The combination of pharmacological innovations with psychotherapeutic approaches leads to the best possible results in the treatment of mood disorders. This principle also applies to treatment with GLP-1 receptor agonists (GLP-1 RA). Essential support for patients starting GLP-1 RA treatment comes from behavioral therapy providers such as psychologists, therapists and specialist nurses who help patients with lifestyle changes, emotional adjustments and adherence challenges. Research studies show that behavioral interventions along with GLP-1 RA therapy help patients maintain lifestyle changes while treating eating disorders and improving their ability to control mood and metabolism [108]. Structured psychotherapeutic support for patients with mood disorders reinforces the mood and quality of life benefits of GLP-1 RAs by helping patients adhere to their treatment plan and promoting behavioral activation and reduction in weight- or metabolism-related avoidance or demoralization. Future clinical care models need to develop integrated approaches that combine GLP-1 RAs with personalized behavioral interventions. The implementation of such multidisciplinary approaches would optimize both metabolic and psychiatric outcomes while creating a more comprehensive, patient-centered model of care. This approach may maximize the impact of GLP-1 RAs by not only targeting metabolic and neurobiological dysfunctions but also reinforcing adherence, behavioral activation, and long-term recovery trajectories. Further exploration of dose–response relationships, long-term maintenance strategies, and the potential preventive role of GLP-1 RAs in high-risk populations (e.g., insulin-resistant youth with depressive symptoms) will be key to translating preliminary findings into clinical practice.

In summary, while GLP-1 RAs hold substantial promise as dual-action agents addressing both metabolic dysfunction and neuropsychiatric symptoms, their integration into psychiatric care demands rigorous clinical trials, biomarker-guided stratification, and mechanistic validation. As research progresses, these agents may help close the gap between psychiatry and metabolic medicine, offering a novel therapeutic avenue for complex mood disorders with a systemic basis.

## 9. Conclusions

The growing recognition of the bidirectional interplay between metabolic dysfunction and mood disorders underscores the need for integrated therapeutic strategies capable of addressing both domains concurrently. GLP-1 RAs, originally developed for glycemic control in type 2 diabetes, have demonstrated mechanistic plausibility and emerging clinical efficacy in modulating key pathophysiological processes implicated in depression, including insulin resistance, neuroinflammation, and reward system dysregulation.

Their pleiotropic effects, targeting both metabolic dysfunction and neuropsychiatric symptoms, position GLP-1 RAs as promising candidates for future therapeutic integration in mood disorders. However, the current evidence base is limited by small sample sizes, heterogeneous study populations, and a paucity of randomized controlled trials in non-diabetic psychiatric cohorts.

Future research should prioritize biomarker-informed, stratified clinical trials that integrate metabolic phenotyping and neuropsychiatric outcome measures. This precision medicine approach may facilitate the identification of subgroups most likely to benefit from GLP-1 RAs and contribute to the development of targeted interventions at the metabolic-psychiatric interface. In conclusion, while GLP-1 RAs are not yet established as standard components of psychopharmacological treatment algorithms, these multimodal properties make GLP-1 RAs a biologically coherent addition to the evolving landscape of psychiatric therapeutics. As our understanding of the metabolic–psychiatric interface deepens, GLP-1 RAs may come to represent a paradigm shift in the management of mood disorders, one that moves beyond symptomatic control to address the systemic basis of treatment resistance.

## Figures and Tables

**Table 1:** Main classes of psychotropic medications, metabolic impact, and effects on mood.

Class/Drug	Primary Receptor Action (Agonist/Antagonist)	Mood-Related Effects	Metabolic Effects/Liability
Second-generation antipsychotics (SGAs)	5-HT2A/2C, H1, M3 receptor antagonists, D2 antagonism/D2 partial agonism	Improve psychotic and mood symptoms; augmentation in depression and bipolar disorder	High risk: weight gain, dyslipidemia, insulin resistance
Olanzapine, Clozapine	Potent H1, 5-HT2A/2C, M3 antagonism	Strong antipsychotic and mood-stabilizing efficacy	Very high metabolic burden (adiposity, glucose dysregulation)
Risperidone, Quetiapine	5-HT2A/2C and D2 antagonism	Effective in bipolar depression and mania	Moderate-to-high risk of weight gain and insulin resistance
Mood stabilizers (Lithium, Valproate)	Multiple targets (e.g., GSK-3 inhibition for lithium; GABAergic facilitation for valproate)	Stabilization of mood swings, relapse prevention	Moderate risk: weight gain, impaired glucose tolerance with long-term use
Antidepressants—TCAs	NE/5-HT reuptake inhibition + H1 and M1 antagonism	Antidepressant effects, anxiolytic properties	Moderate metabolic risk: weight gain, reduced insulin sensitivity
SSRIs (e.g., Paroxetine)	5-HT reuptake inhibition; mild anticholinergic action	Antidepressant efficacy, but higher risk of sedation and fatigue	Mild-to-moderate weight gain, potential reduction in insulin sensitivity
Other SSRIs (e.g., Fluoxetine, Sertraline)	5-HT reuptake inhibition	Antidepressant efficacy, activating profile (esp. fluoxetine)	Neutral or minimal metabolic impact
Adjunctive metabolic agents (Metformin, GLP-1 receptor agonists)	Insulin-sensitizing (Metformin); GLP-1 receptor agonism	Emerging evidence for improved mood, cognition, and treatment response in IR patients	Reduce weight, improve insulin sensitivity, potentially protective
